# The Contribution of Shape Features and Demographic Variables to Disembedding Abilities

**DOI:** 10.3389/fpsyg.2022.798871

**Published:** 2022-03-29

**Authors:** Elisa Morgana Cappello, Giada Lettieri, Andrea Patricelli Malizia, Sonia d’Arcangelo, Giacomo Handjaras, Nicola Lattanzi, Emiliano Ricciardi, Luca Cecchetti

**Affiliations:** ^1^Social and Affective Neuroscience (SANe) group, MoMiLab, IMT School for Advanced Studies Lucca, Lucca, Italy; ^2^Molecular Mind Laboratory, IMT School for Advanced Studies Lucca, Lucca, Italy; ^3^Intesa Sanpaolo Innovation Center SpA, Neuroscience Lab, Torino, Italy; ^4^Laboratory for the Analysis of CompleX Economic Systems, IMT School for Advanced Studies Lucca, Lucca, Italy

**Keywords:** disembedding abilities, embedded figures, visual perceptual abilities, gender, education, shape features, shape symmetry, shape closure

## Abstract

Humans naturally perceive visual patterns in a global manner and are remarkably capable of extracting object shapes based on properties such as proximity, closure, symmetry, and good continuation. Notwithstanding the role of these properties in perceptual grouping, studies highlighted differences in disembedding performance across individuals, which are summarized by the field dependence dimension. Evidence suggests that age and educational attainment explain part of this variability, whereas the role of sex is still highly debated. Also, which stimulus features primarily influence inter-individual variations in perceptual grouping has still to be fully determined. Building upon these premises, we assessed the role of age, education level, and sex on performance at the Leuven Embedded Figure Test—a proxy of disembedding abilities—in 391 cisgender individuals. We also investigated to what extent shape symmetry, closure, complexity, and continuation relate to task accuracy. Overall, target asymmetry, closure, and good continuation with the embedding context increase task difficulty. Simpler shapes are more difficult to detect than those with more lines, yet context complexity impairs the recognition of complex targets (i.e., those with 6 lines or more) to a greater extent. Concerning demographic data, we confirm that age and educational attainment are significantly associated with disembedding abilities and reveal a perceptual advantage in males. In summary, our study further highlights the role of shape properties in disembedding performance and unveils sex differences not reported so far.

## Introduction

Human beings are equipped with a highly efficient visual processing system, which continuously extracts information from complex visual scenes. Along the visual pathway, each image is first decomposed in its essential features, and afterward, single elements are combined to prompt the emergence of a meaningful structure (Malcolm et al., 2016). Indeed, people are naturally prone to perceive in a global manner rather than in single units (*perceptual grouping*; (Koffka, 1935; Brosnan et al., 2004), a disposition influenced by stimulus features such as *proximity, closure, symmetry,* and *good continuation* (Koffka, 1935; Herzog, 2018; see Wagemans et al., 2012a,b for a review).

Over the years, particular attention has been dedicated to understanding which individual differences affect the detection of single elements within the perceptual scene. In this regard, Witkin (1950, 1971) developed the *Embedded Figure Test* (EFT) that requires the subject to spot simple shapes (i.e., *target*) within a more complex geometric pattern (i.e., *embedding context*). In a seminal meta-analysis on sex differences in the processing of spatial features, Linn and Petersen (1985, p. 1484) classified spatial tests in three main categories: *mental rotation, spatial perception, and spatial visualization* tasks. The authors categorize the EFT as a spatial visualization test, as it requires “complicated, multistep manipulations of spatially presented information”. According to Linn and Petersen, other examples of spatial visualization tasks are the Paper Folding test (Shepard and Feng, 1972) and the Block Design, the latter included in the Wechsler Adult Intelligence Scale (Wechsler, 1955) as a measurement of non-verbal IQ.

Interestingly, despite the natural predisposition toward perceptual grouping, performance on EFT showed substantial differences among individuals (Witkin et al., 1962; De-Wit and Wagemans, 2014). According to Witkin et al. (1962) and Witkin (1971), such variability depends on the *field dependence/independence* dimension (FDI), originally defined as the ability to overcome an embedding context. In this theoretical framework, field-dependent individuals are highly influenced by the visual context, whereas someone who is field independent finds it easier to separate specific figure elements from their background. Over the years, Witkin’s work and the EFT drew the attention of several researchers, prompting the study of individual differences in FDI in a variety of contexts, from education and learning performance (Goodenough, 1976; Sadler-Smith and Riding, 1999), to atypical development and autism (Brosnan et al., 2004; Happé and Frith, 2006; Van der Hallen et al., 2015). Previous works also investigated the relationship between disembedding abilities and demographic variables, such as age or sex. Specifically, there is a consensus for the increase of field dependence as a function of age (Lee and Pollack, 1978; Panek et al., 1980; Wiker et al., 2009; Chan and Yan, 2018), which is consistent with the overall decrease in performance on spatial tests observed in the elders (Techentin et al., 2014). Conversely, no significant sex differences were found in EFT performance (Signorella and Jamison, 1978; Linn and Petersen, 1985; Voyer et al., 1995), despite males scoring higher than females on other tests of spatial abilities (e.g., *Mental Rotation Tests*; Linn and Petersen, 1985; Voyer et al., 1995; Baron-Cohen, 2002; Uttal et al., 2013).

Moreover, several doubts concerning the nature of the FDI dimension have been raised (McKenna, 1984). Indeed, the EFT was either defined as a measure of maximum performance (i.e., a *non-verbal ability*) or a propensity (i.e., a *perceptual/cognitive style*). In Witkin’s traditional theoretical framework, FDI is depicted as a preferred way to acquire, organize, manipulate, and interpret information, namely, a cognitive style (Witkin et al., 1962; Kozhevnikov, 2007). However, some authors criticized this interpretation, as cognitive styles should not be assessed using accuracy measurements (McKenna, 1984; Riding and Cheema, 1991; Zhang, 2004). Other authors emphasized the association between performance on the EFT and measures of attention, working memory, and intelligence, as well as a close relationship with learning outcomes and academic achievement (McKenna, 1984; Richardson and Turner, 2000; Evans et al., 2013; Danti et al., 2018). Though, to what extent these findings are consistent across studies is still highly debated (Huygelier et al., 2018) and, despite 60 years of research, the question about which perceptual features prompt individual differences in EFT performance remains unanswered (Milne and Szczerbinski, 2009; Chamberlain et al., 2017; Huygelier et al., 2018).

In this regard, De-Wit and colleagues developed a newer version of the EFT (*Leuven Embedded Figure Test*, L-EFT; De-Wit et al., 2017), in which features that may play a role in perceptual grouping have been intentionally manipulated. By measuring L-EFT performance in two different studies, the authors assessed how these perceptual properties affect embedding effectiveness, making the target more or less difficult to detect. They concluded that target shapes forming *good Gestalts* are easier to spot (e.g., symmetric targets), whereas good continuation between target shape and the embedding context increases task difficulty. However, it is worth noting that both experiments conducted by De-Wit et al. (2017) involved relatively homogeneous samples of undergraduate students with a majority of female individuals (i.e., 87% and 79% for experiments 1 and 2, respectively). This hampered the possibility to assess which personal characteristics are associated with disembedding performance.

Here, we aim to overcome this limitation by investigating the relationship between perceptual stimulus features and L-EFT accuracy in a more heterogeneous group of participants. Three hundred and ninety-five healthy Italian individuals were enrolled in this study, including a comparable number of cisgender men and women, with variable age and years of education. This sample allowed us to measure which stimulus features are processed differently by the two sexes, people of different age groups and education levels.

## Materials and Methods

### Participants

We recruited 395 participants among employees of a large banking group (257F; mean age 39.5 years ± 6.2 standard deviation) from 97 out of the 107 Italian districts. There were no differences in age between male and female individuals (mean age for males: 39.8 ± 6.7 years; mean age for females: 39.3 ± 6 years; Wilcoxon Rank Sum test: z-score = 0.55; *p*-value = 0.58). Two hundred and twenty-three individuals (56.5%) received high-school diplomas (13 years of education), whereas one hundred and seventy-two (43.5%) got a university degree (minimum 16 years of education). There were no significant differences in the number of graduates between the sexes (114 university graduates among women; 58 among men; Chi-square test for independence: *χ*^2^ = 0.2; *p*-value = 0.66). Also, high-school and university graduates did not differ in terms of age (mean age of high-school graduate 39.8 ± 7.5; mean age of college graduate 39.2 ± 4.2).

After excluding four outliers based on the overall accuracy at the L-EFT (see Data analysis for additional details), our final sample was composed of 391 participants (255F; mean age 39.5 years ± 6.2 standard deviation). The two sexes did not differ in terms of age (mean age for males: 39.8 ± 6.7 years; mean age for females: 39.3 ± 6 years; Wilcoxon Rank Sum test: z-score = 0.60; *p*-value = 0.55) and education level (114 university graduates among women; 57 among men; Chi-square test for independence: *χ*^2^ = 0.28; *p*-value = 0.60). Also, no age differences were found between college graduates (43.7%) and people with high-school diploma (56.3%; mean age of high-school graduate 39.8 ± 7.5; mean age of college graduate 39.1 ± 4.1).

All participants gave their consent to take part in the study after risks and procedures were explained. The local Ethical Review Board approved the experimental protocol and procedures (CEAVNO: Comitato Etico Area Vasta Nord Ovest; Protocol no. 1485/2017) and the study was conducted in accordance with the Declaration of Helsinki.

### Stimuli and Experimental Procedure

A 48-trial web-based version of the L-EFT (De-Wit et al., 2017) was employed in our study. In each trial, subjects had to detect a simple shape (i.e., *target*) embedded into a complex pattern of lines (i.e., *context*). Participants were asked to perform the task in a quiet environment free from distraction and not take any break after starting the experiment. For each trial, the target was located in the upper and central part of the canvas, whereas three embedding contexts were presented simultaneously at the bottom of the screen (i.e., leftmost, central, and rightmost part of the canvas). The target shape embedded in the context preserved the same size and orientation as the one at the top of the screen. Only one of the complex figures at the bottom contained the target shape, and its position was randomized across trials. Although response times were not recorded, participants had to choose the context they believed incorporated the target within 60 s since the beginning of each trial. Trials for which a response was not provided within this time limit were regarded as errors. A similar strategy was previously adopted by de-Wit and colleagues (Experiment 2; De-Wit et al., 2017) to reduce the speed-accuracy tradeoff. After providing their answer, participants could not change their responses to previous items. Target shapes may vary on three different parameters: the *vertical symmetry* (i.e., symmetrical vs. asymmetrical figures), the *closure* (i.e., open vs. closed shapes), and the *number of target lines* (i.e., 3, 4, 6, or 8 lines). Instead, the embedding context varied according to the number of lines continuing the target shape (i.e., *number of continued lines*), ranging from 1 to the number of target lines. As compared with the original 64-item version of the L-EFT, we decided to not administer the 16 trials in which the number of continued lines was 0. In De-Wit et al. (2017), these trials were recognized correctly by 96.6% ± 3.3% of participants. Therefore, we excluded these items to reduce completion time without decreasing the sensitivity in the measurement of the disembedding performance.

### Data Analysis

Firstly, we checked for the presence of outliers in the distribution of the overall accuracy and excluded participants based on the generalized extreme studentized deviate test (Rosner, 1983).

To investigate the extent to which stimulus properties and participants’ age, sex, and education influence the performance at a disembedding test, we employed a generalized mixed-effect model (GLMM; Baayen et al., 2008). Participants’ performance at each trial (i.e., correct or wrong answer) represented the dependent binary variable, having a binomial distribution. The link function we selected was the logit function. We modeled *age*, *gender*, *education level*, *number of target lines*, *vertical symmetry*, *closure*, and *number of continued lines* as fixed effects. As De-Wit et al. (2017), among fixed effects, we included the *number of continued lines by vertical symmetry*, the *number of continued lines by closure*, and the *number of continued lines by target lines* interaction terms. Instead, we modeled as random effects participants’ intercept and slope for *vertical symmetry*, *closure*, *number of target lines*, and *number of continued lines*. The *number of target* and *continued lines*, as well as participants’ *age*, were treated as continuous variables, whereas all others were considered categorical. To reduce the possibility of observing false-positive results, the significance threshold (*α* = 0.05) was adjusted for the number of comparisons using the Bonferroni-Holm method (Holm, 1979). As compared to the traditional Bonferroni procedure, this method controls more adequately for type II errors.

All statistical analyses were implemented and performed in Matlab version 2018b (MathWorks Inc., Natick, MA, United States), and the code was made publicly available at the following link: https://drive.google.com/drive/folders/124YksNM0rGdTaxDhevF6VeE4lCn_lZYd?usp=sharing.

## Results

**Figure 1:**
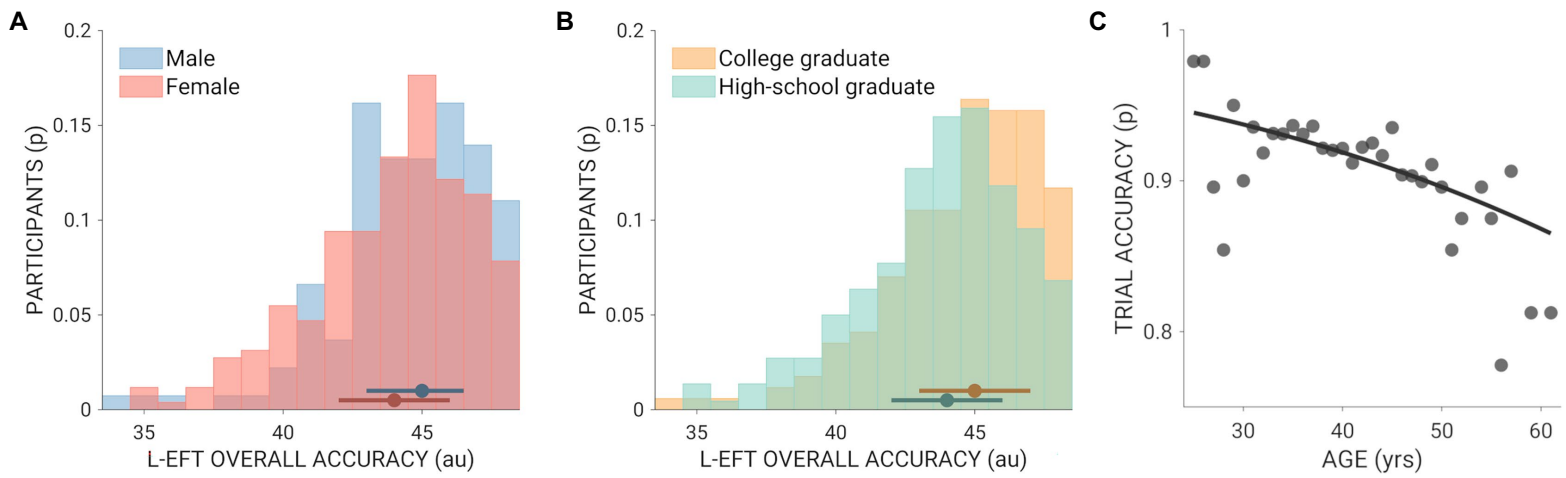
Summarizes the relationship between participants’ demographics and disembedding performance. **(A)** Presents the distribution of the overall accuracy at the L-EFT for males (light blue) and females (light red). **(B)** Shows the overall performance of high-school (light green) and college (light orange) graduates. **(C)** Depicts the proportion of accurate responses at different ages (dark gray dots), as well as the fit of the logit function (solid black line).

Overall, participants exhibited high performance at the L-EFT: average accuracy was 91.89% ± 5.87% standard deviation. All demographic variables considered in the study exerted a significant effect on the disembedding performance. On average, males performed significantly better than females (*F*_(1,18,757)_ = 6.24; *p*-value = 0.01; Bonferroni-Holm adjusted *p*-value = 0.013; Figure 1A), people with a high-school diploma were less accurate than those with a university degree (*F*_(1,18,757)_ = 9.58; *p*-value = 0.002; Bonferroni-Holm adjusted *p*-value = 0.004; Figure 1B), and age was inversely related to disembedding performance (*F*_(1,18,757)_ = 22.37; *p*-value = 2.27^*^10^−6^; Bonferroni-Holm adjusted *p*-value = 1.13^*^10^−5^; Figure 1C).

**Figure 2:**
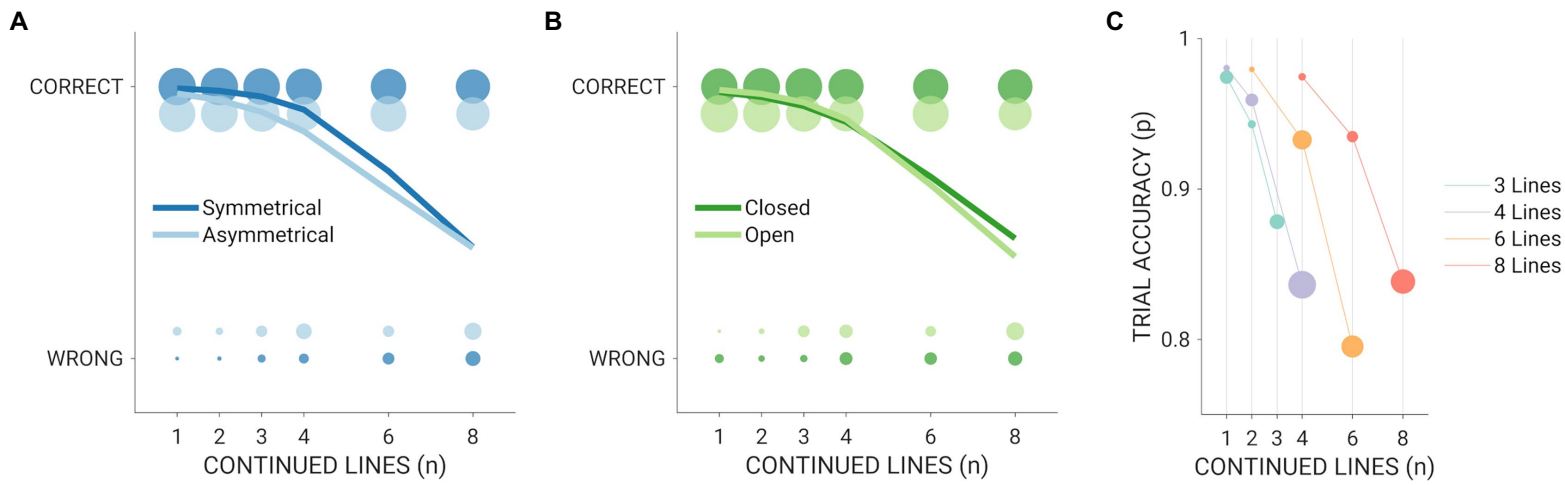
Summarizes the relationship between stimulus properties and disembedding performance. **(A)** Depicts the count of correct and wrong answers (dots) for symmetrical (dark blue) and asymmetrical (light blue) targets at different levels of context complexity (i.e., number of continued lines). The size of the dots reflects the proportion of correct and wrong responses across individuals for a given number of continued lines. Solid lines summarize the fitting of the logit function for the *symmetry by number of continued lines* interaction term. The same plot is presented in **(B)** for closed (dark green) and open (light green) targets. **(C)** Summarizes the extent to which the relationship between the number of target and continued lines influences task accuracy. The size of dots reflects the error rate for each combination of target lines and context complexity (i.e., the larger the dot the higher the proportion of errors). Solid lines represent the fitting of the logit function for the *number of target lines by number of continued lines* interaction term.

When investigating the impact of stimulus properties on task accuracy, we found a significant main effect for the *number of continued lines* (F_(1,18,757)_ = 318.24; *p*-value = 1.34^*^10^−70^; Bonferroni-Holm adjusted p-value = 1.34^*^10^−69^): the higher the number of continued lines, the lower the recognition performance. Symmetrical targets were more easily recognized than asymmetrical ones (*F*_(1,18,757)_ = 118.43; *p*-value = 1.68^*^10^−27^; Bonferroni-Holm adjusted *p*-value = 1.51^*^10^−26^). Of note, the perceptual advantage for symmetrical shapes was not evident when they were embedded in particularly complex (i.e., eight continued lines) contexts, as testified by the significant *vertical symmetry by number of continued lines* interaction term (*F*_(1,18,757)_ = 49.54; *p*-value = 2.01^*^10^−12^; Bonferroni-Holm adjusted *p*-value = 1.41^*^10^−11^; Figure 2A).

Participants’ performance was also higher for open figures than closed ones (F_(1,18,757)_ = 21.27; *p*-value = 4.02^*^10^−6^; Bonferroni-Holm adjusted *p*-value = 1.61^*^10^−5^). However, when inspecting the results of the *closure by number of continued lines* interaction term (*F*_(1,18,757)_ = 25.23; *p*-value = 5.12^*^10^−7^; Bonferroni-Holm adjusted *p*-value = 3.07^*^10^−6^; Figure 2B), we found that closed targets were better recognized than open ones if the embedding context was particularly challenging (i.e., six and eight continued lines); instead, participant’s performance was higher for open shapes embedded in simpler contexts (i.e., one, two, and three continued lines).

As far as the number of target lines is concerned, we found that the higher the shape complexity was, the better the recognition accuracy (*F*_(1,18,757)_ = 19.67; *p*-value = 9.27^*^10^−6^; Bonferroni-Holm adjusted *p*-value = 2.78^*^10^−5^). Importantly, the effect of context complexity in worsening the disembedding performance was greater for more complex shapes (i.e., those with more lines), with the largest drop in accuracy observed for 6-line targets (*number of target lines* by *number of continued lines* interaction term: F_(1,18,757)_ = 76.05; *p*-value = 3.00^*^10^−18^; Bonferroni-Holm adjusted *p*-value = 2.40^*^10^−17^; Figure 2C).

## Discussion

In the current study, we investigated how stimulus properties influence people’s ability to detect simple shapes embedded within complex patterns of lines. A shortened version of the *Leuven Embedded Figure Test* (L-EFT; De-Wit et al., 2017) was used to assess disembedding performance in a sample of healthy Italian individuals, including a comparable number of cisgender males and females, as well as people of different age groups and education levels. Our results show that aging is associated with a reduction in disembedding performance and that, on average, males perform significantly better than females. Also, college graduates show a higher accuracy at the L-EFT compared to those with a high-school diploma.

As for the embedding context properties, the higher the number of continued lines, the worse the recognition performance. We further demonstrate an advantage in the detection of symmetrical, open, and complex shapes. Concerning the interaction between these shape properties and the number of continued lines, we found that if (1) symmetrical and (2) open shapes are embedded in simpler contexts, they are more easily recognized than asymmetrical and closed targets. Moreover, (3) context complexity is increasing task difficulty more for targets with a higher number of lines (i.e., six- and eight-line targets) than for simpler shapes (i.e., three-line targets).

In everyday life, the human visual system continuously organizes disjoint visual inputs into coherent patterns of information that we eventually identify as meaningful objects. This natural predisposition toward perceptual grouping is influenced by a set of stimulus features, such as *symmetry, closure, complexity*, and *good continuation.*

At the beginning of the 20th century, symmetry was described as one of the key grouping principles. When observing a visual scene, elements sharing a symmetric relation tend to be aggregated and perceived as a single figure standing out from the background (Bahnsen, 1928; Koffka, 1935; Machilsen et al., 2009). Symmetry is an extremely salient visual property, as it is a prominent feature in the visual world (e.g., the majority of animals and plants exhibit bilateral symmetry) and a crucial factor for physical attractiveness and mate selection (Rhodes et al., 1998; Hume and Montgomerie, 2001, see Wade, 2010 for a review). According to psychophysical studies, symmetry detection occurs rapidly during pre-attentive stages of perception and does not require a conscious cognitive effort (Treder, 2010; Apthorp and Bell, 2015). Symmetrical shapes are also identified more accurately than asymmetrical figures (Carmody et al., 1977; Machilsen et al., 2009; Pramod and Arun, 2018) and are easier to recall (Attneave, 1955; Kayaert and Wagemans, 2009). Our results extend the advantage of symmetrical shapes to disembedding tasks, as individuals detected more accurately symmetrical targets than asymmetrical ones. In the L-EFT, all the symmetrical target shapes were characterized by vertical symmetry, which may have enhanced the magnitude of the detection advantage in disembedding performance. Indeed, previous studies showed that vertical symmetry is detected more easily than symmetry along other orientations (e.g., *horizontal symmetry*; Wenderoth, 1994; Wagemans, 1995; Evans et al., 2000). Furthermore, we found that the advantage in detecting symmetrical figures was higher when the embedding context was simpler. This evidence is in line with other findings showing how symmetry detection is more difficult in more complex environments, especially when other salient regularities are present (Treder, 2010; Cohen and Zaidi, 2013).

As for symmetry, Gestalt psychologists emphasized the role of closure in perceptual grouping and figure-background segmentation. During the early stages of perception, our visual system automatically groups elements enclosed by a continuous contour, as they are likely to belong to the same physical object (Papale et al., 2018). Closure plays an essential role in shape perception and objects recognition, which are, in turn, critical for building a coherent picture of the external world (Garrigan, 2012). Using a set of visual search experiments, Elder and Zucker (1993) empirically validated the function of closure in bridging 1D closed contours to 2D figures, which represented the boundaries of real-world objects projected onto the retina. Although several studies found a perceptual advantage in closed contours detection (Elder and Zucker, 1993; Kimchi, 2000; Mathes and Fahle, 2007; Garrigan, 2012; Gerhardstein et al., 2012), additional features, such as *element spacing*, *number of turning points*, or *lines length,* might have confounded results relative to *closure* (Pettet et al., 1998; Braun, 1999; Tversky et al., 2004). For instance, Tversky et al. (2004) found only a modest advantage for closed shapes and argued that the facilitation introduced by contour detection could be attributed to the local application of other Gestalt principles (i.e., *good continuation* and *proximity*). More recently De-Wit et al. (2017) investigated disembedding performance at the L-EFT for closed and open target shapes. Across two experiments, they found no differences in closure detection (Experiment 1) or even higher accuracy for open compared to closed shapes (Experiment 2; De-Wit et al., 2017). Our findings support the perceptual advantage for open shapes in disembedding tasks.

In our study, we also focused on the complexity of the target shape. Over the years, complexity was recognized as one important stimulus property affecting individuals’ performance on a broad range of visual tasks. On the one hand, previous works reported that simplicity enhances shape processing, leading to better performances in delayed shape recognition for 2D figures (Kayaert and Wagemans, 2009), in mental rotation tests (Hall and Friedman, 1994), in simultaneous matching, (Pellegrino et al., 1991) and visual search paradigms (Marković and Gvozdenovi, 2001). On the other hand, Mavrides and Brown (1969) explicitly manipulated the redundancy (i.e., nonrandomness; (Donderi, 2006)) in the shape of different polygons, finding that less redundant figures (i.e., more complex shapes) were easier to remember. Likewise, other authors showed that simpler shapes are harder to discriminate (Evans, 1967; De-Wit et al., 2017). A possible explanation comes from Donderi (2006), who claimed that simplicity reduces information content, making figures more similar, thus causing difficulties in recognition. Here, we operationalized shape complexity as the number of target lines related to the number of elements composing the figure (Marković and Gvozdenovi, 2001; Donderi, 2006). Our findings reveal that complex figures are detected better than shapes with intermediate and lower complexity. We further demonstrate that the ability to recognize targets with a relatively higher number of lines (i.e., six and eight) is more affected by context complexity.

Also, that the number of continued lines is inversely related to disembedding accuracy is well explained by the good continuation principle, which postulates that elements aligned to one another tend to be integrated into the same perceptual unit (Wertheimer, 1923). The influence of good continuation on perception has been demonstrated in both infants (Quinn and Bhatt, 2005) and adults (Prinzmetal and Banks, 1977; Pelli et al., 2009). In the L-EFT, good continuation relates to the number of lines starting from the target shape and continuing into the context, thus influencing the effectiveness of targets’ embedding (De-Wit et al., 2017).

Our study also explored the influence of demographic factors on disembedding performance and found that younger individuals score higher than elders. A large body of research supports the existence of a negative correlation between age and performance at different versions of the EFT (Lee and Pollack, 1978; Panek et al., 1980; Wiker et al., 2009; Chan and Yan, 2018). Indeed, due to the physiological deterioration of top-down processing with age (e.g., *working memory*, *speed of processing*, *and inhibitory control*; Park and Reuter-Lorenz, 2009), individuals tend to rely more on external cues, and their ability to filter out environmental information unrelated to the task progressively decreases (Chan and Yan, 2018). Furthermore, it is well documented that a general decline occurs for tasks that require high-level cognitive processing and mental manipulation (Park and Reuter-Lorenz, 2009; Techentin et al., 2014), such as spatial tasks (de Bruin et al., 2016).

The comparison between the performance of men and women in spatial tasks provided mixed evidence across the literature (Maccoby and Jacklin, 1974; Linn and Petersen, 1985; Voyer et al., 1995; Baron-Cohen, 2002; Reber et al., 2004; Hyde, 2005; Andreano and Cahill, 2009; Shepherd and Bar, 2011). For instance Linn and Petersen (1985) found sex differences in *spatial perception* and *mental rotation experiments*, but not in *spatial visualization tasks*, such as the EFT. Conversely, in line with studies in children (Cárdenas and Harris, 2006; Winkielman et al., 2006), our results reveal significant differences in disembedding abilities between the two sexes, with females performing worse than males in a spatial visualization task.

Since the earlier formulation of FDI, Witkin (1976) referred to the educational context as one of the most promising fields for applying his theoretical work. Over the years, several studies highlighted the positive correlation between field independence and students’ achievement on various school subjects (Roszkowski and Snelbecker, 1987; Tinajero and Páramo, 1997; Nicolaou and Xistouri, 2011). In particular, the analytical approach of field-independent people seems more suited to the academic environment, thus promoting learning performance (Kagan and Zahn, 1975; Shade, 1983; Clark and Roof, 1988). Indeed, according to Witkin and Goodenough (1981), field-independent individuals find it easier to organize novel and unstructured information. According to Guisande et al. (2007), field-independent people are also more efficient in inhibiting goal-irrelevant information and maintaining attention to relevant stimuli. Other researchers highlighted the relationship between EFT performance and a wide range of cognitive abilities (e.g., working memory, intelligence; Rittschof, 2010; Evans et al., 2013; Danti et al., 2018), which are known to be related to educational outcomes (Strenze, 2007; Lövdén et al., 2020). Of note, Tinajero and Páramo (1997) observed a significant association between academic achievement and field independence, specifically assessed through EFT. In line with these findings, our results show that individuals holding a university degree scored higher at the L-EFT than those with a high-school diploma. We speculate that, as is the case of other non-verbal abilities (Strenze, 2007; Lövdén et al., 2020), long-lasting education enhances EFT performance by sharpening cognitive strategies and test-taking skills.

The current study presents some limitations that could be addressed in future works. Firstly, our sample is composed of bank employees. Employment is one of the main components of socioeconomic status (SES), which is associated with performance at several cognitive tests (Noble et al., 2005; Farah et al., 2006; Duncan et al., 2012). Thus, occupation and training may have exerted an effect on the association between disembedding performance and sex, age, and education level reported in the current study. However, it is worth mentioning that our participants have been recruited throughout Italy (i.e., 97 out of the 107 Italian districts), encompassing a variety of socio-cultural backgrounds. Such heterogeneity may have compensated for the similarity in participants’ occupation and training.

Secondly, we measured participants’ performance in terms of the proportion of correct answers (i.e., accuracy), without taking into account response time. Previous studies collected and analyzed both measures as they are similarly influenced by task difficulty in perceptual experiments (Palmer et al., 2005). In fact, using the L-EFT, De Wit and colleagues reported no substantial differences between results based on accuracy and those obtained from the analysis of reaction times (De-Wit et al., 2017). Future studies could verify whether the relationship between demographics and disembedding abilities replicates when reaction times are measured.

Thirdly, as participants were not timed we have not been able to perform any analyses related to the speed-accuracy tradeoff. Nevertheless, in our experiment, we imposed a time constraint of 60 s for the participant to respond. A similar strategy was successfully adopted by de-Wit and colleagues (De-Wit et al., 2017) to reduce the speed-accuracy tradeoff (Experiment 2). Thus, we could expect that this effect—if present—marginally impacted our results.

Also, it should be noted that our analyses were limited to perceptual features originally included in the L-EFT (i.e., *vertical symmetry, closure, number of target lines, and number of continued lines*). This set of features is not exhaustive and further studies are needed to explore the influence of other shape features, such as horizontal symmetry, diagonal symmetry, or curvature of boundaries (Chambers et al., 2018), on disembedding performance.

Lastly, it is worth mentioning the relatively small number of trials. As compared with the original 64-item version of the L-EFT, we decided to not administer the 16 trials in which the number of continued lines was 0. Importantly, in De-Wit et al. (2017), these trials were recognized correctly by 96.6% ± 3.3% of participants. Thus, by excluding these items, we reduced completion time without decreasing the sensitivity in the measurement of the disembedding performance.

In summary, our findings highlight that target asymmetry, closure, and good continuation with the embedding context increase task difficulty in a spatial visualization test. Also, although complex targets are recognized better than simpler ones when the embedding context is particularly challenging the reduction in disembedding performance is more evident for shapes with more lines. Moreover, results unveil differences between the sexes for spatial visualization tasks that have not been reported so far and corroborate the association between age, educational attainment, and disembedding performance.

## Data Availability Statement

The datasets presented in this article are not readily available because of privacy concerns and violation of agreement of the informed consent process. Requests to access the datasets should be directed to luca.cecchetti@imtlucca.it.

## Ethics Statement

The studies involving human participants were reviewed and approved by CEAVNO: Comitato Etico Area Vasta Nord Ovest; Protocol No. 1485/2017. The patients/participants provided their written informed consent to participate in this study.

## Author Contributions

AM, Sd’A, NL, ER, and LC planned and supervised the study. EC, GL, GH, and LC processed the experimental data, performed the analysis, drafted the manuscript, and designed the figures. AM, Sd’A, NL, and ER aided in interpreting the results and critically revised the text. All authors contributed to the article and approved the submitted version.

## Funding

The research was conducted under a cooperative agreement between IMT School for Advanced Studies Lucca, Intesa Sanpaolo Innovation Center S.p.A., and Intesa Sanpaolo banking group. This work was supported by Progetto di Attività Integrate—PAI Project (P0135)—granted by IMT School for Advanced Studies Lucca to LC. The funders had no role in study design, data collection and analysis, decision to publish, or preparation of the manuscript.

## Conflict of Interest

Sd’A was employed by the company Intesa Sanpaolo Innovation Center SpA.

The remaining authors declare that the research was conducted in the absence of any commercial or financial relationships that could be construed as a potential conflict of interest.

## Publisher’s Note

All claims expressed in this article are solely those of the authors and do not necessarily represent those of their affiliated organizations, or those of the publisher, the editors and the reviewers. Any product that may be evaluated in this article, or claim that may be made by its manufacturer, is not guaranteed or endorsed by the publisher.
